# Supplementary material for: Population Genomic Analyses Suggest a Hybrid Origin, Cryptic Sexuality, and Decay of Genes Regulating Seed Development for the Putatively Strictly Asexual Kingdonia uniflora (Circaeasteraceae, Ranunculales)
Source: Int J Mol Sci. 2023 Jan 11;24(2):1451. doi: 10.3390/ijms24021451 (PMC9866071; doi:10.3390/ijms24021451)
Supplement: Supplementary file 1 [file ijms-24-01451-s001.zip › GetSnpSeq.pl.pdf]

```perl
#!/usr/bin/perl
# use strict;
#FL1
#FL2
open(FL0, "$ARGV[0]") || die ;
open(FL1, ">$ARGV[1]") || die ;
open(FL2, ">$ARGV[2]") || die ;

# $haplotypeA='';
# $haplotypeB='';
while (<FL0>){
chomp;
@arr=split(/\t/,$_);
@phase = split (/\|/,$arr[3]);
# print $phase[0],"\t",$phase[1],"\n";
# push (@genotype,$arr[1]);
# push (@genotype,$arr[2]);
# print $genotype[$phase[0]], "\t", $genotype[$phase[1]],"\n";
push (@haplotypeA,$arr[$phase[0]+1]);
push (@haplotypeB,$arr[$phase[1]+1]);
# $haplotypeA = $genotype[$phase[0]];
# $haplotypeB = $genotype[$phase[1]];
# print FL1 $haplotypeA;
# print FL2 $haplotypeB;
}
print FL1 @haplotypeA,"\n";
print FL2 @haplotypeB,"\n";
```
