# Supplementary material for: Population Genomic Analyses Suggest a Hybrid Origin, Cryptic Sexuality, and Decay of Genes Regulating Seed Development for the Putatively Strictly Asexual Kingdonia uniflora (Circaeasteraceae, Ranunculales)
Source: Int J Mol Sci. 2023 Jan 11;24(2):1451. doi: 10.3390/ijms24021451 (PMC9866071; doi:10.3390/ijms24021451)
Supplement: Supplementary file 1 [file ijms-24-01451-s001.zip › Supplemental Table S2.pdf]

Supplemental Table S2. Information of contigs containing SNPs.

Contig	Length	No. of genes
Contig2655|ref0000096	35620	0
Contig1938|ref0000500	17953	0
Contig2702|ref0000539	70525	0
Contig2109|ref0001040	16744	0
Contig743|ref0001070	28563	0
Contig459|ref0001171	47723	0
Contig906|ref0001547	31211	0
Contig241|ref0001784	20428	0
Contig2187|ref0002270	69956	0
Contig838|ref0002343	72870	0
Contig2460|ref0002513	91273	0
Contig1887|ref0002835	127470	0
Contig996|ref0002853	15754	0
Contig2091|ref0000088	28543	0
Contig770|ref0000374	56529	0
Contig1737|ref0000925	45667	0
Contig2102|ref0001035	39074	0
Contig1698|ref0001107	19337	0
Contig131|ref0001290	7026	0
Contig37|ref0001686	220100	0
Contig118|ref0001741	30237	0
Contig482|ref0001827	35257	0
Contig1471|ref0001907	39391	0
Contig2188|ref0002262	28275	0
Contig637|ref0002356	21234	0
Contig1759|ref0001362	65423	0
Contig1532|ref0002508	59457	0
Contig2479|ref0000139	36677	0
Contig1043|ref0002899	27378	0
Contig2121|ref0001195	54188	0
Contig903|ref0001552	28090	0
Contig569|ref0000249	52950	0
Contig525|ref0002401	54296	0
Contig1788|ref0001597	97031	0
Contig2369|ref0001924	177591	0
Contig1997|ref0000608	55619	0
Contig2887|ref0002273	56210	0
Contig2391|ref0002841	17145	1
Contig154|ref0000862	182471	0
Contig1512|ref0002747	84085	0
Contig2458|ref0000585	84371	0
Contig2304|ref0001488	36070	1
Contig2196|ref0002087	27921	0
Contig2237|ref0002290	15017	0
Contig375|ref0002916	146824	0
Contig2041|ref0000338	13207	0
Contig210|ref0001117	58597	0
Contig1156|ref0002135	45543	0
Contig2614|ref0001013	17381	0

Contig729|ref0002668	206837	0
Contig2880|ref0000148	16385	0
Contig2026|ref0000807	12076	0
Contig2302|ref0001482	45444	0
Contig2582|ref0001009	287578	1
Contig1704|ref0000256	16045	1
Contig2672|ref0000566	97542	0
Contig1467|ref0000019	116410	0
Contig1958|ref0001860	23490	0
Contig557|ref0000466	38093	0
Contig897|ref0000657	32673	0
Contig2906|ref0000767	40284	0
Contig1866|ref0000006	34055	0
Contig234|ref0002512	17156	0
Contig43|ref0000134	36065	0
Contig2280|ref0000363	40362	2
Contig1649|ref0000901	49468	0
Contig1481|ref0001194	172721	3
Contig141|ref0001064	31240	0
Contig2434|ref0001889	113446	0
Contig2378|ref0001712	17336	0
Contig2885|ref0001855	29094	2
Contig2090|ref0000089	52968	0
Contig1749|ref0001132	30367	0
Contig25|ref0001478	17330	0
Contig2361|ref0001920	44008	0
Contig1181|ref0002378	38746	0
Contig331|ref0000884	31856	0
Contig2789|ref0001906	28454	4
Contig253|ref0001983	33454	0
Contig1027|ref0001294	22472	0
Contig1522|ref0002339	21133	0
Contig534|ref0002211	27184	0
Contig399|ref0001183	28468	2
Contig2771|ref0002074	24844	0
Contig2882|ref0002260	33906	1
Contig418|ref0000281	38323	0
Contig504|ref0002752	29838	0
Contig1048|ref0002906	42946	0
Contig2679|ref0000562	28019	1
Contig2804|ref0001670	94038	0
Contig1035|ref0000079	46499	0
Contig1670|ref0000220	34025	0
Contig499|ref0002006	39149	0
Contig1004|ref0000868	36861	1
Contig1356|ref0000974	38073	0
Contig1723|ref0000988	274383	2
Contig100|ref0001941	41061	0
Contig1184|ref0002373	39673	0
Contig1957|ref0001642	31129	0
Contig2160|ref0002247	34128	0

Contig1445|ref0002568	44377	0
Contig798|ref0000471	220548	4
Contig204|ref0000914	51126	0
Contig2154|ref0000347	56888	0
Contig864|ref0001315	21248	0
Contig1621|ref0001196	49386	1
Contig1763|ref0001579	171663	0
Contig1837|ref0001856	37896	0
Contig2463|ref0002511	46717	0
Contig1346|ref0000752	35719	5
Contig1330|ref0002201	140481	2
Contig2795|ref0001700	36645	1
Contig1577|ref0001808	44187	1
Contig2062|ref0002827	139667	0
Contig1256|ref0000260	40995	0
Contig2446|ref0000349	27664	0
Contig17|ref0001245	24281	0
Contig2576|ref0001724	29563	5
Contig2860|ref0001871	83856	3
Contig983|ref0000147	49458	0
Contig552|ref0000463	24358	1
Contig1933|ref0000504	27167	2
Contig336|ref0000887	44884	0
Contig2597|ref0001492	44124	1
Contig2471|ref0000127	54990	0
Contig1977|ref0001386	28533	0
Contig26|ref0001475	41091	0
Contig2695|ref0002229	50467	1
Contig2769|ref0002258	58586	0
Contig2751|ref0002432	43360	1
Contig899|ref0000655	36804	0
Contig1898|ref0002582	38297	0
Contig2781|ref0001914	37546	0
Contig508|ref0002756	50657	1
Contig876|ref0001524	61236	1
Contig221|ref0002553	39572	1
Contig796|ref0000485	36142	1
Contig260|ref0001355	47626	3
Contig2916|ref0000526	72878	2
Contig901|ref0002777	89342	2
Contig170|ref0000388	36533	1
Contig164|ref0000607	53566	0
Contig2020|ref0000813	65996	2
Contig289|ref0002485	61469	0
Contig2095|ref0000084	75282	0
Contig913|ref0001332	79920	0
Contig908|ref0001542	55673	1
Contig672|ref0001620	53171	4
Contig2642|ref0002813	33247	1
Contig2858|ref0000991	53414	1
Contig2077|ref0000110	72002	7

Contig2284|ref0000367	134180	2
Contig2910|ref0000524	65976	0
Contig1410|ref0001660	17645	0
Contig1925|ref0000277	46815	2
Contig484|ref0001829	41778	0
Contig628|ref0002199	196958	1
Contig2612|ref0001019	91854	4
Contig861|ref0001318	52404	0
Contig2636|ref0001448	48621	0
Contig721|ref0001544	60558	0
Contig248|ref0001781	118940	0
Contig954|ref0000382	95136	0
Contig1910|ref0000943	77313	0
Contig1853|ref0002384	56326	0
Contig2398|ref0002844	56279	4
Contig2207|ref0001743	487064	4
Contig1145|ref0001969	86111	0
Contig1162|ref0001536	81305	2
Contig1062|ref0002312	49561	10
Contig1968|ref0001160	81629	0
Contig1953|ref0001650	84824	0
Contig2195|ref0002090	40758	0
Contig2867|ref0002623	75995	0
Contig2406|ref0002467	44417	0
Contig1948|ref0001653	200078	0
Contig2225|ref0002111	257088	0
Contig2236|ref0002291	97044	0
Contig187|ref0000178	118032	9
Contig891|ref0000663	71854	7
Contig1794|ref0001821	77532	0
Contig49|ref0000124	67576	0
Contig46|ref0000137	63861	3
Contig2754|ref0002427	71466	0
Contig370|ref0002921	62583	0
Contig733|ref0002491	92461	5
Contig1903|ref0000731	76262	5
Contig1194|ref0002538	93105	1
Contig828|ref0002909	71711	8
Contig2319|ref0001259	59038	0
Contig481|ref0001824	52394	2
Contig1974|ref0001385	78981	0
Contig1780|ref0001602	82439	2
Contig1436|ref0002037	57391	0
Contig2411|ref0002648	120270	2
Contig2448|ref0000341	119587	0
Contig2785|ref0001910	59212	1
Contig1094|ref0001986	75685	2
Contig2856|ref0000997	96402	7
Contig1342|ref0000748	100136	5
Contig352|ref0002523	108649	0
Contig3|ref0000573	82060	0

Contig1614|ref0001793	44626	0
Contig717|ref0002153	114425	2
Contig1518|ref0002908	111237	9
Contig1549|ref0001993	112722	0
Contig2269|ref0002859	116055	0
Contig150|ref0000866	131530	0
Contig389|ref0002346	594329	5
Contig2841|ref0000777	88705	3
Contig2508|ref0001718	65526	0
Contig844|ref0002029	121547	0
Contig1601|ref0002926	100605	8
Contig2565|ref0001933	53286	4
Contig1459|ref0002413	64344	1
Contig1235|ref0000712	77294	9
Contig1905|ref0000725	83334	0
Contig1344|ref0000754	80422	1
Contig2432|ref0001885	93062	0
Contig2772|ref0002071	110825	0
Contig137|ref0001288	95566	0
Contig2675|ref0000571	87497	0
Contig1932|ref0002639	81370	12
Contig851|ref0001090	60179	3
Contig2316|ref0001263	92636	0
Contig1973|ref0001390	102162	4
Contig1842|ref0002548	91432	3
Contig1513|ref0002746	98034	0
Contig1107|ref0001099	101129	0
Contig1371|ref0001420	141171	3
Contig1918|ref0000949	77517	0
Contig2759|ref0002436	135885	0
Contig2609|ref0000798	110208	8
Contig627|ref0002188	110109	3
Contig2346|ref0002304	128989	0
Contig612|ref0002715	80387	8
Contig2111|ref0001694	105049	0
Contig98|ref0002779	126779	4
Contig1216|ref0001139	123531	5
Contig1966|ref0001152	89273	7
Contig2451|ref0000592	116400	0
Contig1899|ref0002584	119263	7
Contig196|ref0002894	118301	11
Contig2483|ref0001242	99777	0
Contig730|ref0002494	99250	0
Contig143|ref0001066	92431	2
Contig1014|ref0000648	132836	7
Contig1109|ref0001105	143140	5
Contig2686|ref0002051	123830	5
Contig739|ref0002495	104027	0
Contig517|ref0002560	126198	5
Contig1047|ref0002903	149856	6
Contig1294|ref0002682	126126	5

Contig2079|ref0000102	133231	0
Contig2305|ref0001487	138494	0
Contig2278|ref0000152	102839	7
Contig1042|ref0002900	93020	0
Contig1242|ref0000487	97294	2
Contig801|ref0001759	138870	4
Contig1646|ref0002044	145998	0
Contig378|ref0002913	90893	1
Contig2098|ref0000091	128192	2
Contig857|ref0001092	146595	0
Contig1528|ref0002333	150001	5
Contig379|ref0002912	127077	0
Contig2762|ref0002253	123939	0
Contig528|ref0002392	126364	0
Contig2073|ref0000106	133927	0
Contig687|ref0001308	132611	7
Contig1550|ref0002162	189919	0
Contig2309|ref0001489	81796	0
Contig309|ref0000674	131787	2
Contig360|ref0000195	99298	7
Contig1130|ref0000892	117475	5
Contig2862|ref0001869	149110	8
Contig419|ref0000282	119499	3
Contig14|ref0001248	135862	0
Contig1553|ref0002159	136071	1
Contig2815|ref0001875	162802	7
Contig2314|ref0001261	119424	0
Contig670|ref0001622	184323	9
Contig882|ref0000413	122858	0
Contig1370|ref0001421	123966	2
Contig2019|ref0001495	166016	12
Contig1221|ref0000933	180088	2
Contig1026|ref0001295	121070	11
Contig450|ref0001168	458780	5
Contig2826|ref0001201	135192	0
Contig2038|ref0001029	168491	11
Contig920|ref0001061	150333	11
Contig1976|ref0001387	193243	0
Contig1347|ref0000753	162519	0
Contig1686|ref0001345	94678	9
Contig59|ref0000351	171855	11
Contig993|ref0002848	268273	0
Contig368|ref0000203	183478	28
Contig394|ref0000842	213794	6
Contig1009|ref0001909	178547	0
Contig2827|ref0001202	215167	4
Contig1792|ref0001819	171555	5
Contig2849|ref0000781	134715	6
Contig2546|ref0000606	144327	0
Contig1091|ref0001991	134941	0
Contig497|ref0002002	187907	0

Contig995|ref0002854	188979	0
Contig2696|ref0002232	188313	5
Contig2520|ref0002635	196668	0
Contig270|ref0001570	154017	2
Contig2264|ref0002864	163882	5
Contig1217|ref0001140	207781	0
Contig31|ref0001688	145883	0
Contig2756|ref0002429	195574	3
Contig764|ref0000551	207961	0
Contig1716|ref0002601	178697	0
Contig965|ref0001081	222383	3
Contig2550|ref0002583	185171	11
Contig2510|ref0002103	210019	6
Contig1337|ref0002194	203427	5
Contig2409|ref0002464	136575	0
Contig1421|ref0002216	220527	0
Contig2323|ref0001046	171618	0
Contig1046|ref0002904	200230	0
Contig1504|ref0001645	208304	7
Contig602|ref0002521	165135	0
Contig2681|ref0002058	177626	0
Contig799|ref0000470	223350	0
Contig1003|ref0001932	210734	16
Contig285|ref0002479	197865	0
Contig2796|ref0001701	186015	11
Contig435|ref0000739	209063	15
Contig2524|ref0002631	160112	4
Contig1297|ref0002685	229512	7
Contig594|ref0001719	219701	10
Contig2515|ref0002100	227847	0
Contig94|ref0002783	235759	12
Contig300|ref0000671	175566	0
Contig2375|ref0001706	272968	11
Contig2656|ref0000099	204960	0
Contig2266|ref0002866	344252	0
Contig1070|ref0000187	247422	7
Contig1017|ref0000647	203339	15
Contig2517|ref0002098	222248	8
Contig1669|ref0000432	227694	17
Contig2619|ref0001010	192866	20
Contig1310|ref0001850	235032	0
Contig2562|ref0001928	231048	0
Contig2099|ref0000090	233788	9
Contig1401|ref0001861	240285	11
Contig669|ref0001407	234735	25
Contig477|ref0001613	248119	14
Contig745|ref0001058	189916	0
Contig2835|ref0001434	247676	12
Contig282|ref0002482	206902	23
Contig1525|ref0002338	231911	5
Contig561|ref0000245	216783	0

Contig2056|ref0000280	223374	24
Contig856|ref0001091	222324	14
Contig2525|ref0002630	233194	19
Contig407|ref0000053	207382	20
Contig56|ref0000358	212697	14
Contig2055|ref0000283	258357	0
Contig2775|ref0002078	274451	0
Contig1828|ref0000966	236207	0
Contig426|ref0000513	304088	11
Contig1446|ref0002571	248633	7
Contig598|ref0001361	264153	0
Contig242|ref0001783	268624	8
Contig18|ref0001256	292588	3
Contig1316|ref0001848	322731	0
Contig1096|ref0001984	258558	11
Contig400|ref0000048	281473	0
Contig2311|ref0001266	289466	9
Contig653|ref0002010	281969	0
Contig384|ref0002349	320480	18
Contig575|ref0000011	286237	36
Contig680|ref0001307	289547	35
Contig2214|ref0002184	346351	0
Contig1805|ref0000445	266918	9
Contig1237|ref0000710	297576	8
Contig2742|ref0002616	634732	10
Contig73|ref0000820	292321	0
Contig1382|ref0000207	293801	10
Contig2248|ref0002456	410313	0
Contig1222|ref0000936	360102	0
Contig1045|ref0002901	319395	26
Contig2045|ref0000334	296382	0
Contig2726|ref0000552	273585	7
Contig1591|ref0001382	292754	43
Contig2602|ref0000803	312198	0
Contig1163|ref0001537	362360	9
Contig2063|ref0002828	290068	21
Contig223|ref0002555	274434	0
Contig716|ref0002154	331901	0
Contig1881|ref0001039	527983	13
Contig2540|ref0002519	360094	7
Contig107|ref0001940	270540	0
Contig639|ref0002364	291878	0
Contig895|ref0000659	332323	15
Contig1841|ref0002545	376366	0
Contig2596|ref0000788	287010	0
Contig2902|ref0000763	341986	19
Contig727|ref0001573	359545	20
Contig1517|ref0002733	354271	0
Contig2250|ref0002641	355888	0
Contig546|ref0000693	339844	15
Contig2869|ref0000318	337243	16

Contig2666|ref0000323	392325	14
Contig507|ref0002755	392596	0
Contig172|ref0002300	395782	11
Contig1029|ref0001302	298599	0
Contig2000|ref0001276	313176	0
Contig2691|ref0002233	295153	21
Contig2922|ref0001186	358744	0
Contig2534|ref0002444	353374	0
Contig1249|ref0000496	401312	0
Contig1660|ref0000439	362277	21
Contig1539|ref0002498	343443	0
Contig2058|ref0000294	437319	0
Contig4|ref0000580	422092	8
Contig1870|ref0002741	372961	0
Contig591|ref0002826	300633	0
Contig2545|ref0002529	437305	0
Contig1279|ref0002765	362703	8
Contig529|ref0002393	413702	28
Contig1616|ref0002719	682830	9
Contig1276|ref0002770	423432	13
Contig985|ref0000141	372505	0
Contig588|ref0001553	342936	9
Contig2440|ref0000343	374313	0
Contig658|ref0002585	443559	0
Contig619|ref0002708	354571	0
Contig2719|ref0000316	366459	11
Contig976|ref0002880	374395	0
Contig1243|ref0000486	453330	15
Contig1350|ref0000968	382136	27
Contig2351|ref0002127	416979	7
Contig700|ref0002323	439478	0
Contig246|ref0001787	396098	47
Contig2799|ref0001693	403212	21
Contig585|ref0001561	354050	0
Contig1413|ref0001659	439833	24
Contig2897|ref0000546	471288	26
Contig738|ref0002496	443817	22
Contig293|ref0002728	401264	0
Contig2521|ref0002634	469696	13
Contig1136|ref0000898	438321	7
Contig186|ref0000177	384139	15
Contig1121|ref0000678	580343	0
Contig2438|ref0002299	423026	19
Contig659|ref0002586	409442	66
Contig299|ref0002718	370004	0
Contig1740|ref0000461	493363	28
Contig1795|ref0001820	469206	0
Contig1393|ref0000430	483741	0
Contig462|ref0001400	532057	24
Contig568|ref0000248	409724	18
Contig103|ref0001944	441820	0

Contig621|ref0002190	488670	0
Contig794|ref0000483	486919	25
Contig1565|ref0001593	563644	16
Contig1648|ref0000902	475981	0
Contig1748|ref0001131	531222	0
Contig2847|ref0000775	421986	0
Contig398|ref0000830	492809	28
Contig514|ref0002563	470309	15
Contig1661|ref0000438	513173	24
Contig1303|ref0001633	417459	16
Contig714|ref0002152	570306	27
Contig566|ref0000238	568097	40
Contig2676|ref0000570	532532	38
Contig7|ref0000577	619872	0
Contig866|ref0001313	431790	0
Contig2552|ref0001395	506715	0
Contig2317|ref0001264	478080	0
Contig1721|ref0000986	539176	22
Contig521|ref0002397	525939	0
Contig1676|ref0000214	560633	15
Contig24|ref0001477	538882	25
Contig11|ref0001251	498529	21
Contig324|ref0000222	571538	0
Contig1168|ref0001530	569921	18
Contig2421|ref0000537	534320	0
Contig825|ref0002181	678577	0
Contig533|ref0002208	952946	0
Contig666|ref0001417	489744	0
Contig1673|ref0000219	443943	53
Contig2539|ref0002439	602135	0
Contig1523|ref0002340	640301	29
Contig96|ref0002781	461406	11
Contig1717|ref0002600	691766	0
Contig1106|ref0001098	753018	0
Contig2116|ref0001696	655136	42
Contig579|ref0000023	628683	0
Contig1783|ref0001601	593657	21
Contig1254|ref0000258	684189	0
Contig55|ref0000359	569696	56
Contig2404|ref0002465	628743	0
Contig1488|ref0001277	281122	13
Contig1117|ref0001326	89330	0
Contig1383|ref0000208	687451	21
Contig2779|ref0002080	1289429	0
Contig1992|ref0000292	573517	24
Contig923|ref0001060	578713	36
Contig2593|ref0000793	584220	32
Contig354|ref0002525	557421	0
Contig147|ref0000062	520453	52
Contig10|ref0001252	960239	0
Contig1313|ref0001851	738950	25

Contig1768|ref0001584	713041	0
Contig1988|ref0002410	701036	0
Contig1476|ref0002762	699924	0
Contig559|ref0000459	664744	28
Contig1023|ref0001298	653095	0
Contig1282|ref0002886	582485	43
Contig2888|ref0002788	658626	0
Contig981|ref0000145	564360	72
Contig696|ref0002658	584172	0
Contig1624|ref0000498	611637	53
Contig1190|ref0002534	626166	26
Contig1896|ref0000112	634898	0
Contig1566|ref0001590	749612	0
Contig1278|ref0002764	963295	0
Contig2190|ref0002085	665650	47
Contig1423|ref0002214	678198	40
Contig2029|ref0000816	797656	19
Contig1128|ref0000685	670964	0
Contig1116|ref0001327	642490	47
Contig515|ref0002562	759949	17
Contig968|ref0000161	744377	0
Contig1779|ref0001800	713764	42
Contig153|ref0000863	700964	0
Contig1540|ref0002466	823775	29
Contig372|ref0002919	663938	17
Contig237|ref0000928	682496	26
Contig1724|ref0000980	607841	40
Contig2784|ref0001911	761734	0
Contig2893|ref0000554	795639	23
Contig1851|ref0002386	757475	0
Contig999|ref0002856	580138	0
Contig1767|ref0001583	673859	29
Contig91|ref0002786	654745	0
Contig320|ref0000233	753006	48
Contig23|ref0001472	861454	0
Contig1756|ref0000057	776932	58
Contig1703|ref0000251	946148	28
Contig551|ref0000464	761072	0
Contig1133|ref0000893	566096	14
Contig180|ref0000179	755368	39
Contig725|ref0002675	668376	0
Contig2171|ref0002062	743835	0
Contig1900|ref0000728	749681	44
Contig1480|ref0001197	848836	21
Contig461|ref0001401	800665	33
Contig2143|ref0001903	700063	0
Contig655|ref0002012	762714	0
Contig2006|ref0001282	959912	0
Contig2859|ref0000990	757087	37
Contig1524|ref0002337	781462	0
Contig1253|ref0000265	759290	0

Contig297|ref0002732	828265	0
Contig2388|ref0000114	960012	27
Contig708|ref0002329	876291	0
Contig144|ref0001067	834841	0
Contig409|ref0000044	1175874	34
Contig405|ref0000051	833755	0
Contig2447|ref0000350	738312	0
Contig345|ref0001754	901023	0
Contig2296|ref0000622	812066	42
Contig1098|ref0001994	939002	36
Contig707|ref0002326	853370	0
Contig288|ref0002486	745673	85
Contig2141|ref0001901	884582	0
Contig2709|ref0000544	776533	0
Contig2745|ref0002621	953362	0
Contig2553|ref0001446	907056	0
Contig363|ref0000198	787169	40
Contig156|ref0000860	884667	0
Contig8|ref0000582	989830	0
Contig2022|ref0000811	896688	0
Contig2475|ref0000875	1090960	33
Contig1288|ref0002896	859658	0
Contig2461|ref0002514	957766	0
Contig1764|ref0001580	814031	0
Contig2505|ref0002276	1072167	35
Contig656|ref0002013	1170600	0
Contig2724|ref0000072	920967	0
Contig2594|ref0000790	872034	41
Contig2101|ref0001032	1336507	23
Contig791|ref0000478	937291	0
Contig233|ref0002460	1059193	0
Contig138|ref0001285	980594	35
Contig353|ref0002522	1029928	0
Contig2321|ref0001044	814966	0
Contig1039|ref0001080	928248	47
Contig2776|ref0002075	958938	34
Contig1727|ref0000984	904409	35
Contig2753|ref0002434	1068503	24
Contig2259|ref0002644	1071688	23
Contig119|ref0001740	962628	64
Contig317|ref0000418	932776	59
Contig1752|ref0001791	1004000	0
Contig2765|ref0002254	1044429	0
Contig1557|ref0002163	899880	21
Contig667|ref0001416	1148550	0
Contig1185|ref0002374	1019717	23
Contig2857|ref0000996	1030621	34
Contig2263|ref0002863	1071004	0
Contig614|ref0002709	1107397	22
Contig340|ref0001749	1045082	37
Contig1289|ref0002895	900470	67

Contig1722|ref0000987	1097259	31
Contig2094|ref0000085	1148876	0
Contig2682|ref0002055	976320	0
Contig2502|ref0002283	1006138	0
Contig688|ref0001555	1017699	53
Contig2925|ref0001191	977029	0
Contig564|ref0000240	1248575	0
Contig1454|ref0002404	1124757	0
Contig1695|ref0001110	995586	70
Contig1906|ref0000726	1019149	0
Contig2474|ref0000876	1001083	0
Contig2347|ref0002303	1128405	24
Contig243|ref0001782	1331197	0
Contig1911|ref0000942	990629	42
Contig683|ref0001304	1121475	64
Contig1503|ref0001643	900019	0
Contig305|ref0000668	1005443	28
Contig2|ref0000574	903165	0
Contig2506|ref0002279	1056106	59
Contig2135|ref0001210	1225066	0
Contig2422|ref0000530	1001685	69
Contig218|ref0001125	1281624	54
Contig2002|ref0001278	998040	113
Contig1199|ref0002541	1280407	41
Contig971|ref0002873	1108179	52
Contig932|ref0000856	1334729	0
Contig1362|ref0001181	961086	82
Contig310|ref0000421	1153947	0
Contig1706|ref0001457	1319843	56
Contig1530|ref0002506	1011566	0
Contig281|ref0002483	1033304	54
Contig711|ref0002147	1133039	51
Contig422|ref0000517	1167678	40
Contig145|ref0001068	1361254	31
Contig2728|ref0000533	1268195	37
Contig1141|ref0001965	1121853	0
Contig2620|ref0001219	1088770	61
Contig2128|ref0002391	1501347	35
Contig973|ref0002875	1192929	0
Contig609|ref0002517	1169792	0
Contig892|ref0000660	1082485	70
Contig850|ref0001089	1214705	0
Contig597|ref0001350	1246895	50
Contig1936|ref0000509	1113925	0
Contig2640|ref0002815	1152889	59
Contig2417|ref0002650	1323992	31
Contig1975|ref0001384	1197097	52
Contig2838|ref0001433	1298455	0
Contig2848|ref0000780	1192064	42
Contig2291|ref0000615	1243491	69
Contig148|ref0000058	1399247	0

Contig2230|ref0001747	1272827	0
Contig1024|ref0001297	1214856	0
Contig1608|ref0002923	1128027	0
Contig2665|ref0000322	1413001	0
Contig2472|ref0000130	1119722	0
Contig1963|ref0001157	1126084	0
Contig1196|ref0002536	1336897	52
Contig664|ref0001415	1223002	0
Contig2373|ref0000771	1304068	0
Contig636|ref0002355	1424799	0
Contig2783|ref0001912	1416940	58
Contig1701|ref0000253	1428958	0
Contig184|ref0000175	1120738	119
Contig1873|ref0002744	1221447	92
Contig893|ref0000661	1299653	69
Contig671|ref0001623	1125857	80
Contig1158|ref0002143	1435643	33
Contig1030|ref0001071	1468687	0
Contig1244|ref0000493	1300832	28
Contig1969|ref0001161	1389901	52
Contig685|ref0001310	1518582	0
Contig2630|ref0001454	1054181	139
Contig2844|ref0000772	1356515	0
Contig2504|ref0002277	1184942	141
Contig2333|ref0000834	1117067	127
Contig1730|ref0000924	1447928	79
Contig269|ref0001358	1196095	111
Contig2096|ref0000083	1474436	97
Contig2516|ref0002097	1428900	0
Contig779|ref0002285	1448991	42
Contig2543|ref0001926	1337741	102
Contig2298|ref0000624	1687851	48
Contig160|ref0000640	1463581	49
Contig2130|ref0001213	1674566	43
Contig277|ref0001565	1570555	0
Contig1250|ref0000262	1530683	71
Contig2023|ref0000812	1501876	74
Contig2124|ref0002418	1336315	39
Contig1893|ref0002591	1496266	0
Contig2831|ref0001438	1584694	0
Contig2332|ref0000833	1189343	0
Contig2930|ref0000982	1324674	76
Contig2300|ref0001484	1436243	0
Contig1999|ref0002556	1501224	48
Contig496|ref0002001	1642287	40
Contig2536|ref0002446	1709907	36
Contig2134|ref0001209	1609672	0
Contig2850|ref0000995	1620201	64
Contig252|ref0001982	1456898	77
Contig1962|ref0001156	1685667	0
Contig16|ref0001246	1718906	0

Contig21|ref0001474	1500274	0
Contig1538|ref0002499	1717159	0
Contig2356|ref0002132	1929256	0
Contig2246|ref0002448	1697354	0
Contig2325|ref0001048	1565428	92
Contig1320|ref0002015	1575664	30
Contig1118|ref0001321	1723998	73
Contig2770|ref0002073	1714288	70
Contig1298|ref0002686	1698800	67
Contig613|ref0002716	1502663	172
Contig2837|ref0001436	1657856	0
Contig2657|ref0000098	1817158	0
Contig1612|ref0002723	1646916	95
Contig381|ref0002354	1605454	0
Contig1728|ref0000717	1639803	0
Contig1956|ref0001644	1658271	0
Contig414|ref0000290	1819778	0
Contig2202|ref0001738	1583370	0
Contig2444|ref0002296	1892359	65
Contig1797|ref0001822	1916784	0
Contig603|ref0002520	1876670	71
Contig1428|ref0002224	1561476	118
Contig2030|ref0001025	1773527	112
Contig879|ref0001527	1682802	81
Contig127|ref0001508	2016826	0
Contig1248|ref0000497	1664696	99
Contig1120|ref0000677	1543045	0
Contig2320|ref0001045	1984249	0
Contig1373|ref0001418	1753306	63
Contig740|ref0000171	1598844	52
Contig2395|ref0002837	2064681	67
Contig1195|ref0002537	2494347	0
Contig9|ref0000581	1862849	0
Contig2120|ref0002414	2118665	0
Contig1482|ref0000473	1792486	0
Contig1930|ref0000503	1556632	0
Contig1296|ref0002684	2027245	0
Contig2258|ref0002645	1952506	0
Contig1526|ref0002335	2498417	0
Contig2082|ref0002804	1644651	74
Contig957|ref0000379	1864406	0
Contig1037|ref0001078	1581059	64
Contig2021|ref0000814	1993050	0
Contig2523|ref0002632	1769933	106
Contig1261|ref0000029	2099369	0
Contig40|ref0000131	1637749	0
Contig1006|ref0000870	1708930	123
Contig1710|ref0002595	1886258	124
Contig369|ref0000204	1999883	58
Contig152|ref0000864	1792263	0
Contig692|ref0002662	1658111	139

Contig390|ref0001270	1957087	81
Contig524|ref0002400	1764884	118
Contig1054|ref0002695	1913154	62
Contig1623|ref0001214	1681171	0
Contig715|ref0002151	1682711	0
Contig542|ref0000697	1982980	0
Contig2664|ref0000321	1815245	68
Contig64|ref0000599	1824325	157
Contig2755|ref0002428	1755840	53
Contig2473|ref0000129	1895385	0
Contig2152|ref0001174	2087647	71
Contig1495|ref0001444	2089537	0
Contig1124|ref0000681	1810283	0
Contig1032|ref0001073	2113568	0
Contig2814|ref0001876	1998146	0
Contig1665|ref0000434	1894524	150
Contig1790|ref0001817	1939036	125
Contig1980|ref0002317	2109454	117
Contig2005|ref0001281	2070210	76
Contig712|ref0002150	2226453	0
Contig2507|ref0002278	2100441	74
Contig1365|ref0001176	1822322	0
Contig1995|ref0000611	1815313	0
Contig0|ref0000576	1832933	0
Contig1800|ref0000450	2132932	97
Contig1708|ref0001466	1829850	0
Contig1203|ref0001370	2007485	122
Contig2722|ref0000078	1651247	0
Contig162|ref0000638	2304336	70
Contig1814|ref0000227	2139882	89
Contig2626|ref0001225	2212703	0
Contig2894|ref0000908	2178577	0
Contig986|ref0000142	2255286	99
Contig2218|ref0002146	2142472	103
Contig2810|ref0000989	2214725	153
Contig1309|ref0001638	2349888	85
Contig2035|ref0001020	2487517	0
Contig833|ref0002105	2357887	0
Contig695|ref0002657	2693286	98
Contig2492|ref0001462	2600919	138
Contig2478|ref0001929	2364647	103
Contig2559|ref0001275	2318716	185
Contig2165|ref0002242	2805473	67
Contig209|ref0000909	2560382	0
Contig501|ref0002749	2768538	98
Contig1668|ref0000433	2350273	0
Contig1159|ref0002142	2770261	0
Contig2829|ref0001200	2742525	217
Contig2589|ref0001000	2879601	111
Contig1926|ref0000274	2861216	0
Contig1642|ref0002048	2668262	113

Contig247|ref0001786	2697503	0
Contig1631|ref0002368	2781057	0
Contig630|ref0002361	2838310	0
Contig2241|ref0002455	3097619	90
Contig1658|ref0000683	2809023	134
Contig816|ref0001954	3130047	106
Contig2711|ref0001870	3042805	97
Contig1519|ref0002907	2303650	0
Contig1810|ref0000231	2866924	130
Contig157|ref0000859	2727513	0
Contig1438|ref0002033	3188244	82
Contig1991|ref0000295	3111116	0
Contig2791|ref0000757	3072570	0
Contig1558|ref0002158	3061608	135
Contig1579|ref0001810	3231187	103
Contig2011|ref0001503	3432469	109
Contig2245|ref0002451	2994898	110
Contig371|ref0002920	3273727	121
Contig1450|ref0002408	3238893	104
Contig195|ref0002722	3189232	0
Contig1493|ref0001429	2961086	177
Contig265|ref0001715	3044142	0
Contig1002|ref0001936	3194487	202
Contig1472|ref0002758	3215882	0
Contig1238|ref0000719	2981183	0
Contig463|ref0001399	3229337	109
Contig2426|ref0000543	3224334	87
Contig1777|ref0001792	2710893	0
Contig510|ref0000612	3076795	159
Contig1|ref0000575	3526998	98
Contig1835|ref0001834	3992156	0
Contig1871|ref0001872	3699471	0
Contig1457|ref0002403	3670362	0
Contig836|ref0002093	3138004	0
Contig574|ref0000012	3154921	0
Contig1281|ref0002887	3480323	0
Contig2823|ref0001206	3657587	139
Contig2668|ref0000329	3703665	156
Contig1875|ref0002738	3533489	0
Contig2069|ref0002824	3529192	119
Contig732|ref0002492	2981294	280
Contig1521|ref0002342	3077289	0
Contig2392|ref0002842	3585851	138
Contig216|ref0001123	3571395	0
Contig2340|ref0002310	3939987	0
Contig624|ref0002187	3716331	0
Contig424|ref0000515	3543359	210
Contig2866|ref0001881	3724550	117
Contig303|ref0000670	4087713	221
Contig759|ref0002871	3820484	0
Contig2608|ref0000797	4443625	144

Contig2117|ref0001702	4363817	0
Contig2139|ref0001254	4424270	0
Contig358|ref0002516	3954464	186
Contig1137|ref0000897	4200025	125
Contig690|ref0002660	4132851	151
Contig750|ref0002677	4355316	188
Contig651|ref0002580	4229260	0
Contig2723|ref0000077	4709776	0
Contig2832|ref0001599	4061581	195
Contig1818|ref0000234	4710542	192
Contig2017|ref0001497	4649653	0
Contig548|ref0000689	4685848	239
Contig1363|ref0001182	5301524	194
Contig975|ref0002877	4856599	188
Contig342|ref0001746	5441041	120
Contig835|ref0002092	5126625	186
Contig722|ref0002672	5041616	160
Contig674|ref0001626	5218826	177
Contig1744|ref0000444	5142023	0
Contig1233|ref0000714	4851045	0
Contig1258|ref0000254	5602675	0
Contig126|ref0001507	5101076	0
Contig83|ref0002612	5162191	0
Contig1858|ref0002381	5671722	0
Contig819|ref0001963	5551227	182
Contig2013|ref0001501	5704433	254
Contig2928|ref0000769	5997497	0
Contig229|ref0000457	6219004	0
Contig757|ref0002666	5675505	309
Contig1620|ref0001208	6160183	247
Contig2210|ref0001946	6645983	199
Contig2155|ref0000721	6210988	0
Contig2282|ref0000361	6215404	302
Contig2567|ref0001931	7003029	185
Contig1879|ref0002735	6607739	0
Contig1916|ref0000945	7255963	233
Contig1743|ref0000456	7943970	0
Contig1406|ref0001868	7746464	287
Contig2366|ref0001919	8125951	0
Contig1920|ref0000272	7593296	235
Contig629|ref0002198	7472585	348
Contig1838|ref0000744	8680253	0
Contig337|ref0000886	8893252	0
Contig1392|ref0000431	11531354	360
Contig1867|ref0000005	3246	0
Contig1262|ref0000032	14908	0
Contig2873|ref0000039	23588	0
Contig984|ref0000140	5729	0
Contig1079|ref0000193	30799	0
Contig1944|ref0000301	36729	0
Contig2868|ref0000317	28102	0

Contig2288|ref0000369	3255	0
Contig178|ref0000396	10744	0
Contig1060|ref0000398	26840	0
Contig1065|ref0000401	13668	0
Contig311|ref0000420	25027	0
Contig423|ref0000516	28495	0
Contig898|ref0000654	39907	0
Contig1230|ref0000715	18587	0
Contig2338|ref0000831	16975	0
Contig2103|ref0001034	45108	0
Contig2328|ref0001043	9560	0
Contig859|ref0001084	43014	0
Contig1101|ref0001101	51761	0
Contig1832|ref0001127	1977	0
Contig132|ref0001293	22293	0
Contig1683|ref0001340	40975	0
Contig1379|ref0001426	43122	0
Contig2639|ref0001455	70925	0
Contig2303|ref0001481	13880	0
Contig580|ref0001556	4181	0
Contig673|ref0001621	27141	0
Contig2801|ref0001667	43295	0
Contig2574|ref0001722	4728	0
Contig2572|ref0001728	6522	0
Contig2205|ref0001745	24545	0
Contig249|ref0001780	196690	1
Contig2212|ref0001948	13929	0
Contig2176|ref0002067	42149	0
Contig2876|ref0002118	27700	0
Contig535|ref0002210	19728	0
Contig1529|ref0002334	14177	0
Contig1187|ref0002376	1393	0
Contig2538|ref0002438	47143	0
Contig2532|ref0002442	33971	0
Contig2401|ref0002470	67258	0
Contig2741|ref0002617	25026	0
Contig2254|ref0002637	17941	0
Contig2410|ref0002649	3784	0
Contig751|ref0002671	50979	0
Contig1474|ref0002760	11677	0
Contig2735|ref0002795	6084	0
Contig2644|ref0002819	27666	0
Contig1283|ref0002885	13751	0

No. of repetitive sequences
1
1
1
1
1
1
1
1
1
1
1
1
1
2
2
2
2
2
2
2
2
2
2
2
2
3
3
4
4
5
5
6
6
7
7
8
8
8
9
9
10
10
10
10
10
11
11
12
14

14
15
15
15
16
17
17
18
18
19
19
19
20
20
21
22
23
24
25
25
26
26
27
27
27
27
27
28
28
28
29
29
30
31
31
31
32
32
32
33
33
34
34
34
35
35
35
35
35
36
36

36
37
37
38
38
39
39
39
39
40
40
41
41
41
43
43
43
43
43
44
44
44
45
45
46
46
46
46
46
47
48
48
49
49
50
50
51
51
53
53
54
54
54
54
55
56
56
57
57
58
59

59
59
59
60
60
60
61
61
61
61
61
62
62
62
62
64
64
65
66
67
67
67
68
69
70
70
70
71
71
73
74
75
75
75
76
77
77
77
78
78
79
79
79
81
82
83
83
85
86
86
88

88
89
89
90
90
91
91
92
93
93
93
94
94
95
96
96
96
97
98
99
99
100
100
100
100
101
104
105
108
108
109
109
109
110
112
113
115
115
116
116
116
117
117
119
121
121
122
122
122
122
123

124
124
126
127
128
130
130
130
132
132
132
132
133
133
135
136
137
138
139
140
141
141
142
143
144
145
151
151
152
152
152
155
156
158
158
159
159
159
163
163
164
165
166
172
173
175
176
177
178
180
181

181
182
182
183
184
185
186
186
188
188
189
189
190
191
193
194
195
196
205
206
213
215
216
216
219
221
224
224
225
225
226
228
230
233
233
237
238
240
245
246
246
246
248
249
250
250
251
253
254
255
256

256
256
257
259
260
264
264
267
269
275
279
279
281
282
282
283
290
290
290
293
294
295
296
296
305
306
307
307
308
310
313
316
321
322
322
324
325
330
333
333
338
339
342
348
354
355
355
355
358
359
364

364
364
367
368
371
373
374
378
379
383
384
386
386
389
389
391
392
394
397
404
410
412
413
416
416
420
422
431
432
436
439
447
448
449
465
467
469
472
476
478
481
486
492
494
496
498
502
506
506
508
512

512
516
516
520
521
523
526
531
534
535
538
539
539
543
544
546
548
551
570
576
576
577
585
589
603
603
605
608
613
613
616
619
620
622
640
646
648
652
655
655
660
660
662
662
667
669
671
671
679
681
682

683
706
708
711
713
718
719
723
725
726
726
732
732
734
737
742
743
744
745
748
752
755
756
768
771
772
772
772
773
773
774
780
783
786
798
805
806
806
810
811
812
813
814
818
818
820
820
830
832
832
834

836
838
839
845
852
852
855
857
858
866
867
877
883
887
891
893
897
898
905
905
908
919
921
938
961
965
970
986
986
994
994
996
998
999
999
1001
1005
1012
1015
1032
1034
1035
1035
1039
1041
1045
1053
1058
1059
1064
1078

1079
1082
1086
1086
1092
1094
1096
1101
1104
1109
1111
1113
1114
1132
1133
1136
1139
1142
1142
1151
1157
1160
1161
1161
1165
1176
1187
1188
1191
1196
1201
1204
1207
1214
1216
1216
1218
1219
1224
1228
1229
1233
1236
1241
1245
1246
1251
1255
1256
1261
1267

1282
1290
1293
1296
1302
1302
1325
1333
1338
1345
1349
1354
1356
1357
1367
1375
1379
1381
1382
1385
1394
1397
1400
1406
1416
1423
1426
1442
1465
1466
1475
1505
1505
1511
1512
1522
1522
1554
1557
1560
1566
1569
1573
1580
1591
1599
1617
1619
1620
1639
1647

1650
1664
1693
1711
1716
1720
1728
1735
1736
1737
1742
1753
1756
1758
1767
1769
1773
1779
1790
1802
1808
1809
1825
1829
1839
1840
1841
1848
1856
1859
1863
1873
1874
1875
1880
1890
1897
1898
1901
1913
1914
1926
1951
1953
1963
1964
1981
1981
2012
2018
2025

2031
2031
2040
2043
2053
2054
2065
2065
2068
2072
2078
2081
2082
2089
2089
2096
2110
2113
2117
2119
2153
2171
2198
2209
2226
2247
2256
2327
2344
2348
2387
2404
2423
2428
2440
2453
2464
2488
2569
2600
2623
2670
2693
2694
2751
2766
2789
2799
2801
2809
2843

2853
2881
2904
2953
2957
2958
2963
2981
2987
3028
3043
3061
3061
3062
3099
3102
3133
3133
3154
3161
3204
3210
3249
3295
3302
3339
3369
3393
3397
3487
3497
3501
3507
3513
3577
3588
3608
3609
3625
3631
3666
3708
3767
3805
3832
3905
3995
3999
4044
4128
4132

4201
4249
4316
4321
4366
4478
4487
4653
4774
4871
4938
4986
5125
5128
5149
5197
5272
5292
5328
5396
5501
5596
5616
5775
5784
5866
5891
6077
6194
6317
6515
6669
6693
6940
6949
7430
7819
8054
8061
8100
8177
8631
9469
11410
0
0
0
0
0
0
0

0
0
0
0
0
0
0
0
0
0
0
0
0
0
0
0
0
0
0
0
0
0
0
0
0
0
0
0
0
0
0
0
0
0
0
0
0
0
0
0
0
0
0
