# Supplementary material for: Population Genomic Analyses Suggest a Hybrid Origin, Cryptic Sexuality, and Decay of Genes Regulating Seed Development for the Putatively Strictly Asexual Kingdonia uniflora (Circaeasteraceae, Ranunculales)
Source: Int J Mol Sci. 2023 Jan 11;24(2):1451. doi: 10.3390/ijms24021451 (PMC9866071; doi:10.3390/ijms24021451)
Supplement: Supplementary file 1 [file ijms-24-01451-s001.zip › Supplemental_ Notes_revision.pdf]

DNA preparation

Total genomic DNA (≥10 ug, ≥50 ng/ul) was isolated from fresh leaves using the conventional cetyltriethylammonium bromide (CTAB) method (Doyle and Doyle 1987). DNA degradation and contamination were monitored on 1% agarose gels. DNA purity was checked using the NanoPhotometer® spectrophotometer (IMPLEN, CA, USA). DNA concentration was measured using Qubit® DNA Assay Kit in Qubit® 2.0 Flurometer (Life Technologies, CA, USA).

Library preparation for sequencing

A total amount of 700 ng DNA per sample was used as input material for the DNA sample preparations. Sequencing libraries were generated using NEB Next® Ultra DNA Library Prep Kit for Illumina® (NEB, USA) following manufacturer's recommendations and index codes were added to attribute sequences to each sample. Briefly, the Chip DNA was purified using AMPure XP system (Beckman Coulter, Beverly, USA). After adenylation of 3' ends of DNA fragments, the NEB Next Adaptor with hairpin loop structure were ligated to prepare for hybridization. Then electrophoresis was used to select DNA fragments of a specified length. 3 μL USER Enzyme (NEB， USA) was used with size-selected, adaptor-ligated DNA at 37°C for 15 min followed by 5 min at 95 °C before PCR. The PCR was performed with Phusion High-Fidelity DNA polymerase, Universal PCR primers and Index (X) Primer. At last, PCR products were purified (AMPure XP system) and library quality was assessed on the Agilent Bioanalyzer 2100 system.
