# Supplementary material for: Population Genomic Analyses Suggest a Hybrid Origin, Cryptic Sexuality, and Decay of Genes Regulating Seed Development for the Putatively Strictly Asexual Kingdonia uniflora (Circaeasteraceae, Ranunculales)
Source: Int J Mol Sci. 2023 Jan 11;24(2):1451. doi: 10.3390/ijms24021451 (PMC9866071; doi:10.3390/ijms24021451)
Supplement: Supplementary file 1 [file ijms-24-01451-s001.zip › Supplemental_Figure S1-S3, S5-S6, Table S1, S3, S4_revision.pdf]

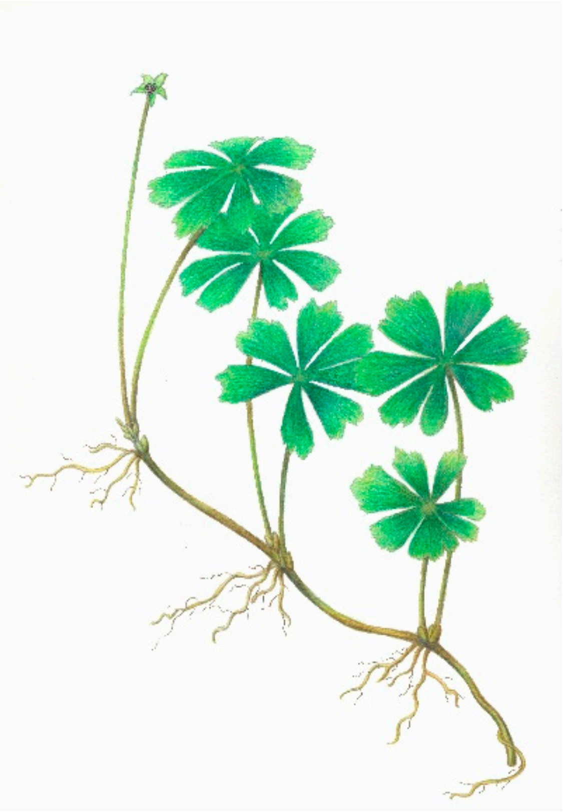

Supplemental Figure S1 Asexual reproduction via rhizome buds observed in *K. uniflora.* New individuals are formed by means of rhizome rupture, which occurs on rhizomes more than three years old.

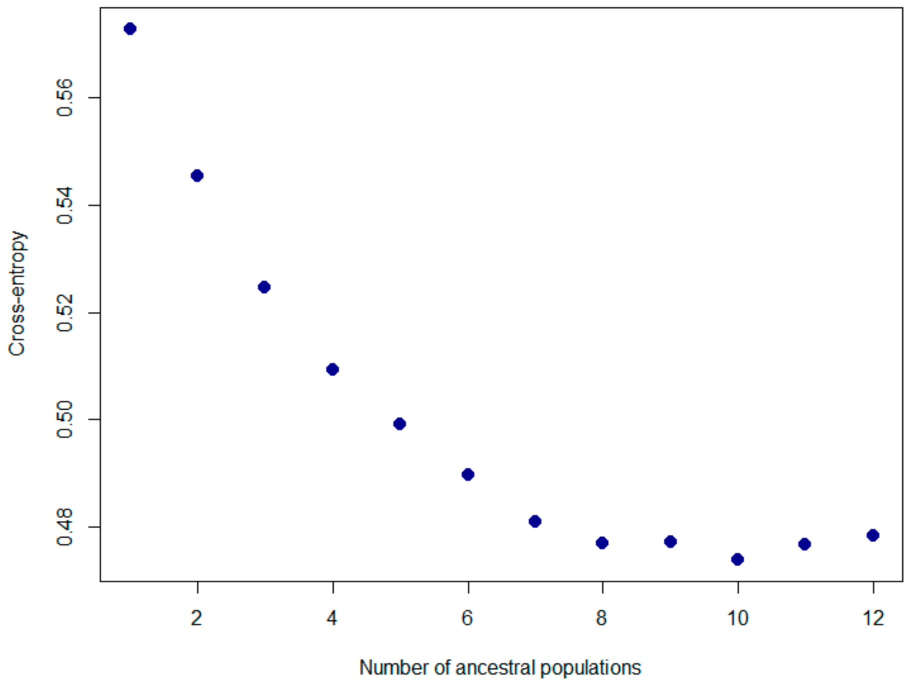

Supplemental Figure S2 Values of the cross-entropy criterion of *K* (2-12) predefined in the LEA (Landscape and Ecological Association) analysis.

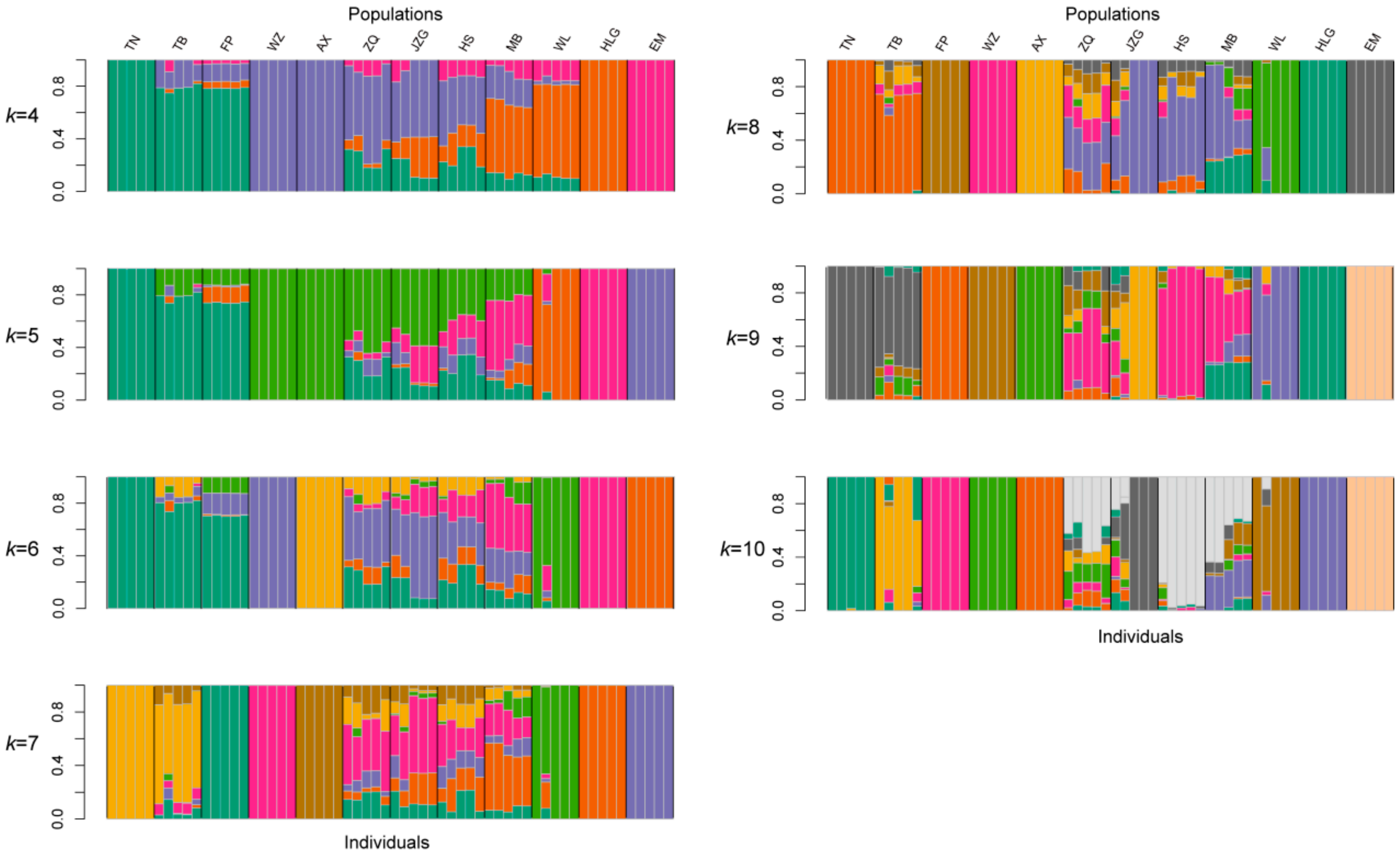

Supplemental Figure S3 Genetic groups of *K. uniflora* inferred by LEA (Landscape and Ecological Association) analysis when setting *K*=4-10.

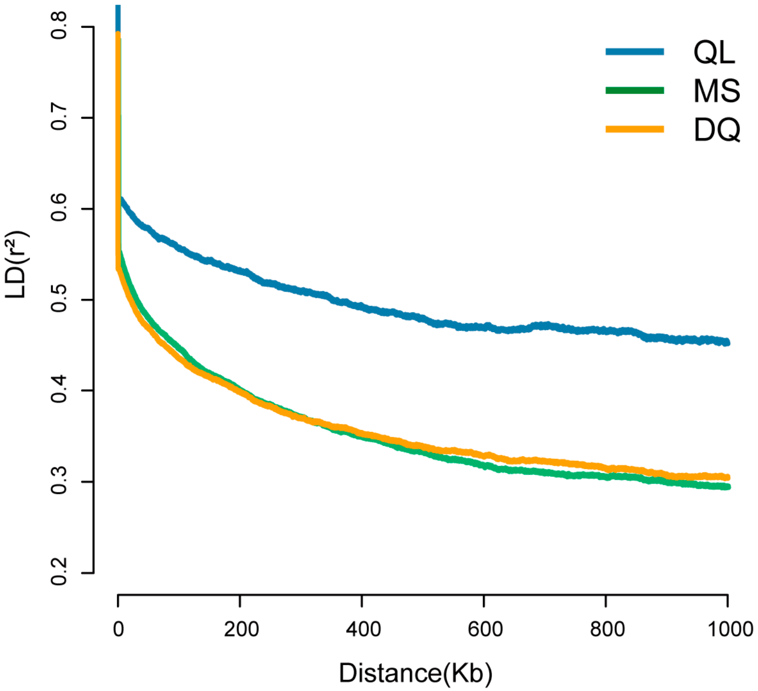

Supplemental Figure S5 Decay of linkage disequilibrium (LD) with physical distance in three genetic groups of *Kingdonia uniflora*. Averages of pairwise linkage disequilibrium measures r^2 are plotted for each bin of distances between pairs of SNPs. The displayed data are for the bins with pairs of SNPs separated by ≤1000 kb

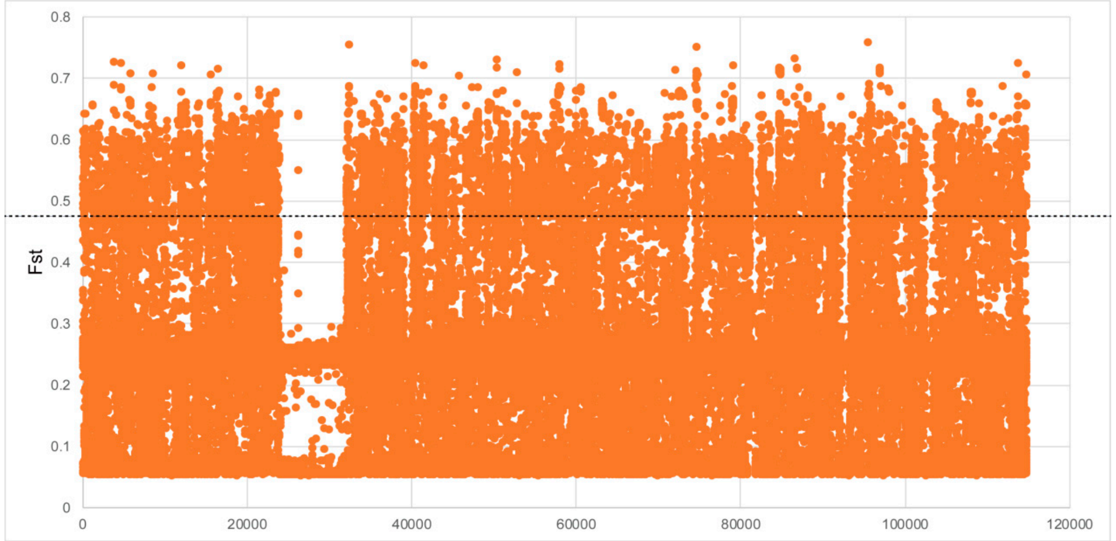

Supplemental Figure S6 F_{ST} values of 1, 114,746 SNPs among 60 *K. uniflora* individuals calculated by BAYESCAN. The x-axis represents the sequence number of SNPs. The black dash line indicates the cut-off line between identified outliers and non-outliers.

Supplemental Table S1. Information of resequencing reads for each individual of *K. uniflora.*

Samples	Raw Reads	Clean Reads	Depths of coverage	Accession number
TN1	51,027,738	50,765,637	34.8	
TN2	46,756,886	46,510,276	31.8	
TN3	43,249,393	43,003,486	29.4	
TN4	40,951,869	40,730,366	27.8	
TN5	35,584,733	35,400,146	24.2	
WL1	39,375,744	39,152,228	26.8	

WL3	38,903,937	38,656,346	26.4
WL5	32,546,125	32,354,786	22.2
WL6	40,671,643	40,437,678	27.6
WL7	40,628,675	40,369,729	27.6
ZQ7	39,963,786	39,716,297	27.2
ZQ3	43,615,885	43,338,862	29.6
ZQ5	39,464,945	39,215,846	26.8
ZQ4	42,635,467	42,337,173	29
ZQ1	36,531,497	36,338,066	24.8
TB1	49,077,603	48,767,309	33.4
TB3	36,688,363	36,419,033	24.8
TB5	36,043,511	35,841,078	24.4
TB7	34,603,411	34,404,621	23.6
TB9	33,058,875	32,834,357	22.4
WZ3	32,444,070	32,264,793	22
WZ4	31,827,747	31,640,452	21.6
WZ2	38,015,892	37,806,436	25.8
WZ1	33,340,675	33,165,931	22.6
WZ5	32,249,336	32,098,812	22
AX4	44,142,701	43,819,874	30
AX9	39,948,467	39,686,180	27.2
AX2	46,043,925	45,720,425	31.2
AX7	40,578,295	40,309,818	27.6
AX3	44,243,655	43,939,354	30
HS11	42,256,873	42,000,749	28.8
HS10	38,244,010	37,978,069	26

HS8	44,571,367	44,308,766	30.4
HS6	42,268,519	41,741,309	28.6
HS5	38,612,247	38,343,519	26.2
HLG5	47,973,012	47,710,146	32.6
HLG9	36,452,284	36,221,647	24.8
HLG7	33,623,575	33,423,989	22.8
HLG1	36,023,575	35,809,342	24.4
HLG3	41,629,174	41,390,763	28.4
EM5	38,462,035	38,223,491	26.2
EM3	48,005,189	47,699,822	32.6
EM4	43,659,391	43,388,140	29.6
EM1	46,241,281	45,994,988	31.4
EM2	38,572,766	38,338,167	26.2
JZG8	36,086,631	35,846,991	24.6
JZG9	34,921,693	34,679,138	23.8
JZG10	39,978,799	39,671,802	27.2
JZG11	36,920,939	36,662,409	25
JZG12	40,321,785	40,074,994	27.4
MB7	44,455,962	44,216,766	30.2
MB4	35,638,763	35,419,345	24.2
MB3	32,680,176	32,389,467	22.2
MB1	33,730,641	33,526,920	23
MB2	37,421,816	37,162,369	25.4
FP1	39,533,969	39,370,526	27
FP2	37,436,213	37,258,503	25.4
FP3	37,633,695	37,443,969	25.6

FP4	34,378,288	34,203,388	23.4
FP5	34,930,795	34,759,707	23.8

Supplemental Table S3. Premature stop codons (*) detected in genes related with seed development.

Gene and individual name	Positions of premature stop codons
KingUnif05328.1_MB4	MLK*QIPYFICAGETSWIAKSALSSALSNLNPEDSFSIIAFNGNTYLFSSSLEPATKETI<br>EKAIEWINMNFIAGGGRNRTSGSQAGGHMKPTLSLGTSDIVFTQQVEYGQPMPYLYESRH<br>LHALKRVRGSGGRFVNTKKMEESNDQNASNVGFLQRGHENRTGSEHLQSEVSNAFASATS<br>CFDVNIVSTGDTDLLRQSDLRFRGYPPQ*
KingUnif30787.1_MB1	MAPVSFEQTSKKVCVMDASGRLGSSLVDRLLQRGYIVHAAIQNNGIMNCDNNKKNLRVFR<br>SDPFDYHSIINALRGCSALFYTFEPPQEQPIFDEFTAEEEVRAAHNVLEACAQTETVEKV<br>VFTSSVTAAIWREDRSKSSQDFDERHWSDVNFCRKFKLWHALSKTLAERTAWALAKDRDV<br>SMVSINAGLLLGPELSVKHPYLKGTAEMFEDGVFVTVDLIFLVDAHIAVFEDVSTYGRYF<br>CFNHVINRPEDAVQFAQMLTPSPPPPPPLESCEDSTVHQQRISNKKLNKVMVDYGMVDEE<br>AIEVENDYYQI*RFYHLIMLGEQRRLCLRLLAEDVINEADEEVGVAEEETVAVETEIGNM<br>LPVFRLETCFCYNHISFLEVFYIGGCFRFCYNQQSFLEEVFHVGGADVPELEV*
KingUnif03794.1_TB9	MMDFFRPVLQITTITGPVDICVSDGTLLISCELSISEVNKLQIGSIVRLISTRGFPVHYV<br>GAATHNRQTKSRIQGWDPSTISNLKFHDFTVPPFLNHIPNPNSSDKFPTYLIPTFDAERY<br>LREPLAAFLKTLSQNTRRIVVIRDSLMCFAAEEASFHHNAEAYYFNSCSIFANVYYFWEF<br>IGKPADGGNLIPSYPVGRGSWEGCFNDDFCEAVERNREYMNTKLSTGDLYNTCDPIEGEF<br>MKNLTQHPILGENKNKWAIGPLNPMHLDSKMNQTNKCIKWLNIQSPNLVIYVSFGTMISI<br>SDEQITELALRLERSKQMFIWVLRDADCGDIYAVKEELRKIQLPDGYEDRVEGVGLIIRD<br>WAPQLDILAHSTTGGFMSL*LELLHGEPQHGCTHCSLPHADGSTF*
KingUnif11633.1_WL1	MMDQQGEFKGKVCVTGASGYLASWLVKRLLLSGYQVRGTVRDPGNHGKLKHLWQLEGAKE<br>RLELVKAELMEEGSFDEAIMGCEGVEILDPAIKGTRNVLNSCKKNPSLNRVVLTSSSTAL<br>RTRVDFDSKVPLDESSWSSEDYCQELKVTKKHEDITDV*IGLKQITDVNVVPIDCAHGRY

	LCSSFVLGNNDLVSILAKRYPSLPVPERFEQLDGPDFEFNTSKL*

Supplemental Table S4. All known extant populations of *K. uniflora*. Location information of the sampled *K. uniflora* populations are indicated with bold characters (*n*, number of individuals analyzed).

Group	Population	*n*	Longtitude (*E*)	Latitude (*N*)	Altitude (m)	Voucher specimens information
	**FP**	**5**	**107°48′55″**	**33°42′29″**	**2638**	**Zhang & Zhang19001**
	**TB**	**5**	**107°48′29″**	**34°0′6″**	**3206**	**Zhang & Zhang19002**
**QL**	**TN**	**5**	**107°46′43″**	**33°54′52″**	**3101.5**	**Zhang & Zhang19003**
	**AX**	**5**	**103°33′54″**	**33°45′3″**	**3162**	**Zhang & Zhang19004**
	**HS**	**5**	**102°46′59″**	**32°14′29″**	**3553**	**Zhang & Zhang19005**
	**JZG**	**5**	**103°43′34″**	**33°2′38″**	**3534**	**Zhang & Zhang19006**
	**WZ**	**5**	**103°42′10″**	**33°53′29″**	**3008**	**Zhang & Zhang19007**
**MS**	**ZQ**	**5**	**104°5′28″**	**33°42′13″**	**2758**	**Zhang & Zhang19008**
	**EM**	**5**	**103°19′48″**	**29°31′55″**	**2702**	**Zhang & Zhang19009**
	**HLG**	**5**	**101°58′7″**	**29°33′8″**	**3502**	**Zhang & Zhang19010**
	**MB**	**5**	**102°17′54″**	**31°42′20″**	**3411**	**Zhang & Zhang19011**
**DQ**	**WL**	**5**	**102°34′23″**	**30°30′14″**	**3076**	**Zhang & Zhang19012**
	CQ		107°33′29″	33°13′21″		
	TJH		104°46′3″	32°35′15″		
	SGN		102°49′7″	31°2′6″		
