# Supplementary material for: Population Genomic Analyses Suggest a Hybrid Origin, Cryptic Sexuality, and Decay of Genes Regulating Seed Development for the Putatively Strictly Asexual Kingdonia uniflora (Circaeasteraceae, Ranunculales)
Source: Int J Mol Sci. 2023 Jan 11;24(2):1451. doi: 10.3390/ijms24021451 (PMC9866071; doi:10.3390/ijms24021451)
Supplement: Supplementary file 1 [file ijms-24-01451-s001.zip › Supplemental_Figure S4.pdf]

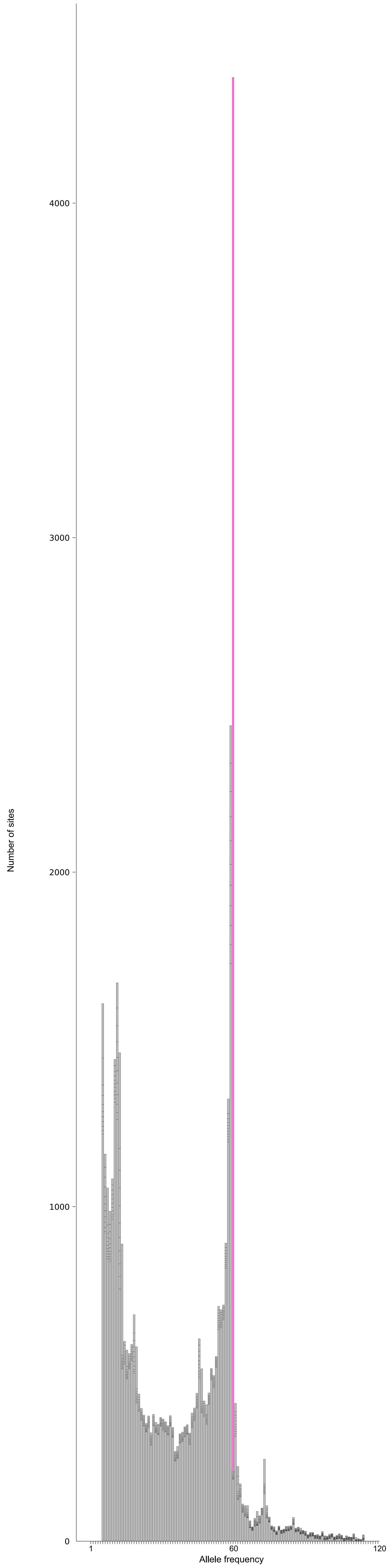

Supplemental Fig. S4 The site frequency spectrum (SFS) depicting the number of sites with different allele frequency across 60 *K. uniflora* individuals (one allele can display a maximum frequency of 120 among 60 diploid individuals). Heterozygous genotypes shared among all 60 individuals are highlighted using the purple color.
