# Supplementary material for: Population Genomic Analyses Suggest a Hybrid Origin, Cryptic Sexuality, and Decay of Genes Regulating Seed Development for the Putatively Strictly Asexual Kingdonia uniflora (Circaeasteraceae, Ranunculales)
Source: Int J Mol Sci. 2023 Jan 11;24(2):1451. doi: 10.3390/ijms24021451 (PMC9866071; doi:10.3390/ijms24021451)
Supplement: Supplementary file 1 [file ijms-24-01451-s001.zip › Supplemental_Methods_batchRenameSeq.pl.pdf]

```perl
#!/usr/bin/perl
# use strict;
open(FL0, "$ARGV[0]") || die ;
open (OUT1, ">$ARGV[1]") || die ;
@list =<FL0>;
chomp @list;
foreach $i (@list){
    print $i,"\n";
    open $fh, "snp2.".$i.".cds.fasta";
#   open $fh1,">","snp2.".$i.".cds.re.fasta";
    while (<$fh>){
        chomp;
            if ($_ =~ /^\>/){
                print OUT1 $_."+".$i,"\n";
#               print OUT1 ">".$i,"\n";
                }
            else {
#               $_ = substr ($_, 0, length ($_)-3);
                print OUT1 $_,"\n";}
                }
}
```
