# Supplementary material for: Population Genomic Analyses Suggest a Hybrid Origin, Cryptic Sexuality, and Decay of Genes Regulating Seed Development for the Putatively Strictly Asexual Kingdonia uniflora (Circaeasteraceae, Ranunculales)
Source: Int J Mol Sci. 2023 Jan 11;24(2):1451. doi: 10.3390/ijms24021451 (PMC9866071; doi:10.3390/ijms24021451)
Supplement: Supplementary file 1 [file ijms-24-01451-s001.zip › Supplemental_Methods_ConnectSeq.pl.pdf]

```perl
#!/usr/bin/perl
# use strict;
#FL1，fasta格式文件
#FL2，序列名称，不含有>符号

open (FL0, "$ARGV[0]") || die ;
open (FL1, ">$ARGV[1]") || die ;
open (FL2, ">$ARGV[2]") || die ;

while (<FL0>){
chomp;
if ($_=~ /^\>/){
    $_ =~ s/\>.*\+(.*)/$1/;
    print FL1 ">".$_."\n";
    push (@list,">".$_."\n");}
else {
    print FL1 $_."\n";
    push (@list,$_."\n");}
}


# @list=<FL0>;
chomp @list;

print $list[0];

for($j=0;$j<@list;$j+=2){
$ref1{$list[$j]}.=$list[$j+1];}


@keys =keys %ref1;
print $keys[1],"\t";

while (my ($k,$v)=each %ref1){print FL2 "$k\n$v\n"}
```
