# Supplementary material for: Population Genomic Analyses Suggest a Hybrid Origin, Cryptic Sexuality, and Decay of Genes Regulating Seed Development for the Putatively Strictly Asexual Kingdonia uniflora (Circaeasteraceae, Ranunculales)
Source: Int J Mol Sci. 2023 Jan 11;24(2):1451. doi: 10.3390/ijms24021451 (PMC9866071; doi:10.3390/ijms24021451)
Supplement: Supplementary file 1 [file ijms-24-01451-s001.zip › Supplemental_Methods_GetNoInterCodonSeq.pl.pdf]

```perl
#!/usr/bin/perl
# use strict;
#FL1，fasta格式文件
#FL2，序列名称，不含有>符号
open(FL0, "$ARGV[0]") || die ;
open(FL1, "$ARGV[1]") || die ;
open(OUT, ">$ARGV[2]") || die ;
open(OUT1, ">$ARGV[3]") || die ;

@inter = <FL0>;
chomp @inter;

@arr1=<FL1>;
chomp @arr1;

for($i=0;$i<@arr1;$i++){
    if ($arr1[$i] =~ /^\>/){
        @tem= split (/\+/, $arr1[$i]);
        $tem[0] =~ s/>(.*)/$1/;
        if ($tem[0] ~~ @inter){
#cds
            $arr1[$i+1] = substr ($arr1[$i+1], 0, length($arr1[$i+1])-3);
#pro
#           $arr1[$i+1] = substr ($arr1[$i+1], 0, length($arr1[$i+1])-1);
            print OUT1 $arr1[$i],"\n",$arr1[$i+1],"\n";}
        else {
#cds
            $arr1[$i+1] = substr ($arr1[$i+1], 0, length($arr1[$i+1])-3);
#pro
#           $arr1[$i+1] = substr ($arr1[$i+1], 0, length($arr1[$i+1])-1);
            print OUT $arr1[$i],"\n",$arr1[$i+1],"\n";}
    }
}
```
