# Supplementary material for: Population Genomic Analyses Suggest a Hybrid Origin, Cryptic Sexuality, and Decay of Genes Regulating Seed Development for the Putatively Strictly Asexual Kingdonia uniflora (Circaeasteraceae, Ranunculales)
Source: Int J Mol Sci. 2023 Jan 11;24(2):1451. doi: 10.3390/ijms24021451 (PMC9866071; doi:10.3390/ijms24021451)
Supplement: Supplementary file 1 [file ijms-24-01451-s001.zip › Supplemental_Methods_GetSampleGenome.sh.pdf]

```bash
#!/bin/bash
list=PathToSampleList/list.txt
ref=PathToGenomeReference/KingdoniaUniflora_genome_scaffolds_V1.0.fasta
GATK=PathToGatk/gatk
vcf=PathToSnp/all.merged.snp.filtered.vcf
gffread=PathToGffread/gffread
gff=PathToGff/KingdoniaUniflora_genome_scaffolds_V1.0.gff
Genelist=PathToGenelist/Genelist

for ((i=1; i<=60; i++))
do
    echo $i;
    sample=$(tail -n +$i $list |head -1| cut -f 1)
    j=`expr $i + 9 `
    cat $vcf | cut -f 1,2,3,4,5,6,7,8,9,$j >snp.filtered.$sample.vcf
    cat snp.filtered.$sample.vcf | grep ^# >snp.filtered.$sample.head
    cat snp.filtered.$sample.vcf | grep -E '1/1|2/2' >snp.filtered.$sample.homo
    cat snp.filtered.$sample.head snp.filtered.$sample.homo >
    snp.filtered.$sample.homo.vcf
    $GATK IndexFeatureFile -I snp.filtered.$sample.homo.vcf 2>$sample.index.log&
    $GATK FastaAlternateReferenceMaker -R $ref -O $sample.remapped.fasta -V
    snp.filtered.$sample.homo.vcf 2>$sample.log &
    perl RenameGenomeChr.pl $sample.remapped.fasta $sample.remapped.rename.fasta &
    $gffread $gff -g $sample.remapped.rename.fasta -x
    $sample.remapped.rename.cds.fasta -y $sample.remapped.rename.pro.fasta
    2>$sample.index.log &
    perl  MutiToOne.pl  $sample.remapped.rename.pro.fasta
    $sample.remapped.rename.pro.oneline.fasta
    perl  MutiToOne1.pl     $sample.remapped.rename.pro.oneline.fasta
    $sample.remapped.rename.pro.oneline.ok.fasta
    sed -i '1d' $sample.remapped.rename.pro.oneline.ok.fasta
    perl  MutiToOne.pl  $sample.remapped.rename.cds.fasta
    $sample.remapped.rename.cds.oneline.fasta
    perl  MutiToOne1.pl     $sample.remapped.rename.cds.oneline.fasta
    $sample.remapped.rename.cds.oneline.ok.fasta
    sed -i '1d' $sample.remapped.rename.cds.oneline.ok.fasta
    perl GetKingSeq.pl $Genelist $sample.remapped.rename.cds.oneline.ok.fasta
    Gene.$sample.cds.fasta
    perl GetKingSeq.pl $Genelist $sample.remapped.rename.pro.oneline.ok.fasta
    Gene.$sample.pro.fasta
done
```
