# Supplementary material for: Population Genomic Analyses Suggest a Hybrid Origin, Cryptic Sexuality, and Decay of Genes Regulating Seed Development for the Putatively Strictly Asexual Kingdonia uniflora (Circaeasteraceae, Ranunculales)
Source: Int J Mol Sci. 2023 Jan 11;24(2):1451. doi: 10.3390/ijms24021451 (PMC9866071; doi:10.3390/ijms24021451)
Supplement: Supplementary file 1 [file ijms-24-01451-s001.zip › Supplemental_Methods_SplitProByStopCodon.pl.pdf]

```perl
#!/usr/bin/perl
# use strict;
#FL1，fasta格式文件
#FL2，序列名称，不含有>符号
open(FL0, "$ARGV[0]") || die ;
open (OUT1, ">$ARGV[1]") || die ;
open (OUT2, ">$ARGV[2]") || die ;


@arr1=<FL0>;
chomp @arr1;

for($i=0;$i<@arr1;$i++){
# @tep=split(/\s/,$arr1[$i]);
# $tep[0] =~ s/^>(.*)\-mRNA.*/$1/;
# $ref1{$tep[0]}=$arr1[$i+1];
    if ($arr1[$i] =~ /\./){
        # push (@inter,$arr1[$i]);
        print OUT2 $arr1[$i-1],"\n",$arr1[$i],"\n";}
    elsif ($arr1[$i] !~ /^\>/){
        # push (@nointer,$tep[0]);
        print OUT1 $arr1[$i-1],"\n",$arr1[$i],"\n";}
}
```
